# Long-read epigenomic diagnosis and prognosis of Acute Myeloid Leukemia

**DOI:** 10.21203/rs.3.rs-5450972/v1

**Published:** 2024-12-12

**Authors:** Jatinder Lamba, Francisco Marchi, Marieke Landwehr, Ann-Kathrin Schade, Vivek Shastri, Matin Ghavami, Fernando Sckaff, Richard Marrero, Nam Nguyen, Vikash Mansinghka, Xueyuan Cao, William Slayton, Petr Starostik, Raul Ribeiro, Jeffrey Rubnitz, Jeffery Klco, Alan Gamis, Timothy Triche, Rhonda Ries, Edwards Anders Kolb, Richard Aplenc, Todd Alonzo, Stanley Pounds, Soheil Meshinchi, Christopher Cogle, Abdelrahman Elsayed

**Affiliations:** University of Florida; University of Florida; University of Florida; University of Florida; University of Florida; Massachusetts Institute of Technology; University of Florida; University of Florida College of Pharmacy; University of Florida; Massachusetts Institute of Technology; University of Tennessee Health Science Center; University of Florida; University of Florida; St. Jude Children’s Hospital; St. Jude Children’s Research Hospital; St. Jude Children’s Research Hospital; Children’s Mercy Hospitals and Clinics; Van Andel Institute; Fred Hutchinson Cancer Center; Leukemia Lymphoma Society; Children’s Hospital of Philadelphia; University of Southern California; St. Jude Children’s Research Hospital; Fred Hutchinson Cancer Research Center; University of Florida; St. Jude Children’s Research Hospital

## Abstract

Acute Myeloid Leukemia (AML) is an aggressive cancer with dismal outcomes, vast subtype heterogeneity, and suboptimal risk stratification. In this study, we harmonized DNA methylation data from 3,314 patients across 11 cohorts to develop the Acute Leukemia Methylome Atlas (ALMA) of diagnostic relevance that predicted 27 WHO 2022 acute leukemia subtypes with an overall accuracy of 96.3% in discovery and 90.1% in validation cohorts. Specifically, for AML, we also developed *AML Epigenomic Risk*, a prognostic classifier of overall survival (OS) (HR=4.40; 95% CI=3.45–5.61; P<0.0001), and a targeted *38CpG AML signature* using a stepwise EWAS-CoxPH-LASSO model predictive of OS (HR=3.84; 95% CI=3.01–4.91; P<0.0001). Finally, we developed a specimen-to-result protocol for simultaneous whole-genome and epigenome sequencing that accurately predicted diagnoses and prognoses from twelve prospectively collected patient samples using long-read sequencing. Our study unveils a new paradigm in acute leukemia management by leveraging DNA methylation for diagnostic and prognostic applications.

## Introduction

Acute Myeloid Leukemia (AML) is a devastating disease associated with high morbidity and mortality. Decades of fundamental research demonstrate that leukemic stem cells are highly heterogeneous, elusive, and ever evolving, so translating that many layers of complexity into clinical care has been a monumental challenge^1^. Compromised epigenomic integrity hallmarks the disease in the young and the old, which warrants comprehensive rather than isolated study. Diagnostic subtyping and risk stratification are foundational pillars of treatment regimens, clinical trial design, and systematically inform therapy decisions. However, despite extensive evidence pointing to the central role of DNA methylation in marking proliferation and differentiation of AML^2–4^, current guidelines rely on a selective list of genomic lesions for diagnosis and prognosis, which does not capture disease heterogeneity and pleiotropy. Due to a lack of feasible technological solutions, implementation of epigenomics in clinical hematology/oncology settings are lacking, and so are studies providing evidence to its promise.

Genomic and epigenomic analyses are plagued by the overwhelming amount of data they generate, outputting an odyssey of variants of unknown significance, obfuscating clinically relevant information. To provide a refined and impactful data with meaningful interpretation, novel machine learning algorithms have shown success in detecting patterns invisible to human curation. Seminal work in recent years demonstrated the use of dimension reduction techniques to define diagnoses of central nervous system tumors and sarcomas^5,6^, which led to WHO recommending genomic classification systems over morphology despite full awareness that most clinicians only currently have access to the latter^7^. To address that, long-read DNA sequencing technologies now allow for detection of cytosine modifications natively, which enables concomitant genome and epigenome sequencing^8,9^. In this study, we present a robust framework that combines large-scale epigenomic data harmonization, machine learning, web-interface, and innovative sequencing technologies to advance the diagnostic and prognostic landscape of AML. Our findings support the integration of epigenomic profiling into the hematology/oncology unit setting, offering a pathway toward more personalized and effective trial designs and treatment modalities for patients with AML.

## RESULTS

**Figure 1:**
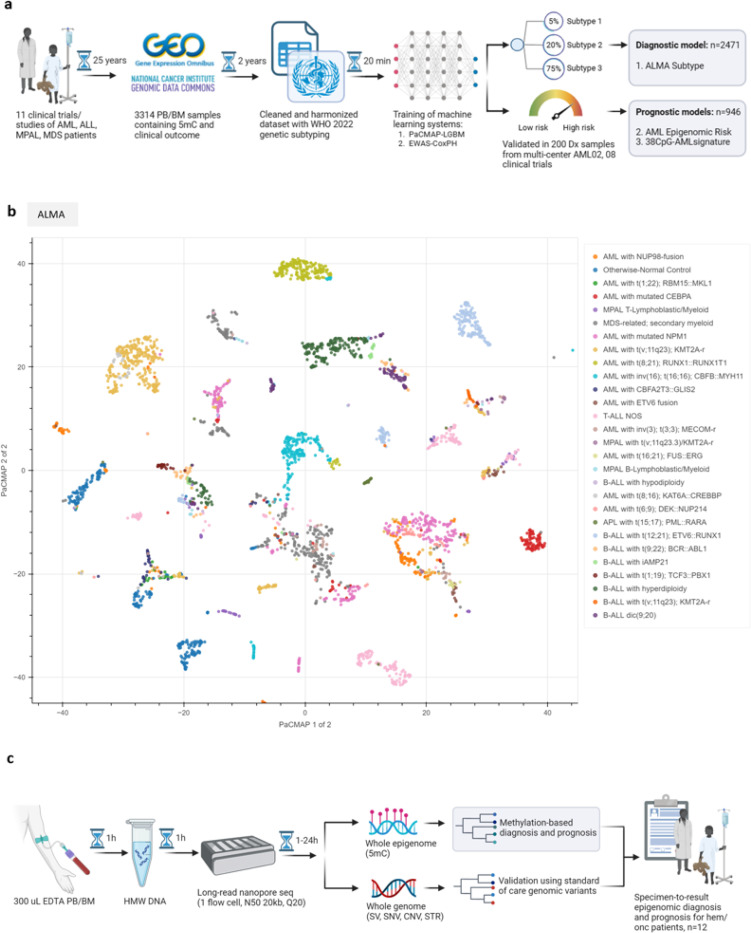
The Acute Leukemia Methylome Atlas Study. **A)** Overall design of computational models. **B)** Acute Leukemia Methylome Atlas (ALMA) defined by dimension reduction algorithm PaCMAP in the overall population. Each data point is a patient sample deriving from bone marrow or peripheral blood at diagnosis, relapse, or remission. As an unsupervised model, only 5mC values of 331,556 CpGs were used to define the clusters. **C)** Clinical implementation of long-read nanopore sequencing pipeline. AML, acute myeloid leukemia; ALL, acute lymphoblastic leukemia; MDS, myelodysplastic syndrome; MPAL, mixed phenotype acute leukemia; APL, acute promyelocytic leukemia; PaCMAP, pairwise controlled manifold approximation; WHO, World Health Organization; PB, peripheral blood; BM, bone marrow; EDTA, Ethylenediaminetetraacetic acid; HMW gDNA, high molecular weight genomic DNA.

The overall study schema and details of study design that used Pairwise Controlled Manifold Approximation (PaCMAP) as a machine learning tool are summarized in Figure 1A and available as an interactive web-app at https://f-marchi.github.io/UF-LambaLab-ALMA-app/.

### The Acute Leukemia Methylome Atlas (ALMA) Maps Hematopoietic Heterogeneity.

#### Overall acute leukemia patient representation:

Our PaCMAP based ALMA diagnostic model compresses methylation values of 331,556 CpGs into two dimensions for visualization and five dimensions for subtype and risk classification. Samples with similar epigenomes clustered together, globally representing the following cell populations of origin: AML (n=1221), ALL (n=700), MDS-related or secondary myeloid neoplasms (MDS-like) (n=223), Acute promyelocytic leukemia (APL) (n=31), mixed phenotype leukemia (MPAL) (n=48), and otherwise-normal control (n=251). These were further subdivided into local structures that overlapped with the heterogeneous clinical subtypes within the disease domains described above (Figure 1B, Supplementary Table S1 and S2).

### ALMA accurately stratifies WHO2022 subtypes by DNA methylation.

#### Diagnostic model performance per class:

**Figure 2:**
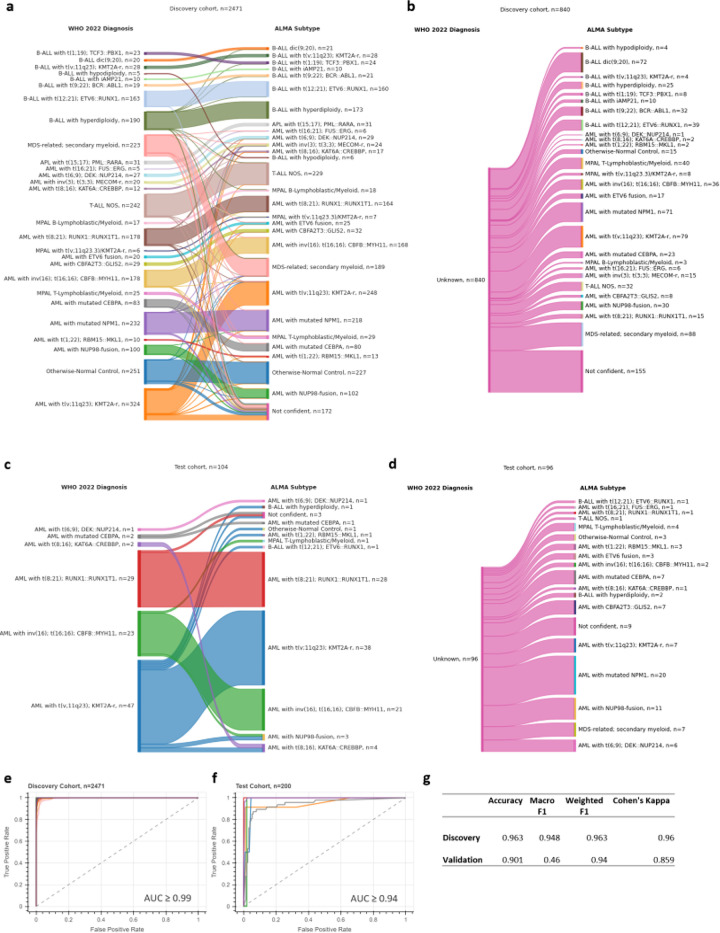
ALMA Subtype classification according to WHO 2022 subtypes. Sankey diagram displaying discovery cohort comparison between WHO 2022 diagnosis (left) and ALMA Subtype (right) for samples with **A)**or without **B)** WHO 2022 clinical annotation available. The width of the bands indicates the number of patient samples in each category. Comparison applied to validation cohort describing samples with **C)** or without **D)** WHO 2022 annotation available. **E)**Receiver operating characteristic (ROC) curves for the discovery cohort (n=2471) and **F)** validation cohort (bottom, n=200). The curves depict the true positive rate against the false positive rate for model predictions. Specific per-class AUC results are available in the electronic notebook. **G)**Table summarizing classification performance metrics: accuracy, macro F1, weighted F1, and Cohen’s Kappa for the training and test cohorts, indicating the overall predictive performance.

The diagnostic model within ALMA named ALMA Subtype is a LightGBM supervised multi-class classifier that uses five PaCMAP coordinates to predict 27 WHO 2022 subtypes plus otherwise-normal control (Supplementary Fig 1A, B). Applying these subtypes to the discovery dataset with available WHO 2022 diagnosis annotation (n=2471) generated a per class 5-fold cross validation (CV) concordance score ranging from 0.87 for *AML with t(v;11q23); KMT2A-r* and 0.93 for *MDS-related, secondary myeloid*, both of which highly heterogeneous, ambiguous subtypes, to a perfect 1.00 in seventeen other categories (Figure 2A, Supplementary Fig 1C). The annotated validation cohort (n=104) only contained AML samples and showed per class concordance scores of 0.83 for *AML with t(v;11); KMT2A-r* (n=47), 0.91 for AML with *inv(16); t(16;16); CBFB::MYH11* (n=23), 1.00 for *AML with mutated CEBPA* (n=2), 1.00 for *AML with t(6;9;DEK::NUP214* (n=1), 1.00 for AML with t(8;16); KAT6A::CREBBP (n=2), 1.00 for AML with *t(8;21); RUNX1::RUNX1T1* (n=29) (Figure 2B, Supplementary Fig 1D).

#### Subtype classification in undefined WHO22 samples:

Perhaps unintuitively but powerfully, inclusion of ALL, APL, MDS, and control samples enhanced the overall capacity of the classifier to predict specific AML subtypes. Notably, the resulting classifier allowed prediction of clinical diagnosis of 840 samples in the discovery cohort and 96 samples in the validation cohort for which WHO 2022 diagnosis annotation was unavailable (Figure 2C,D). These samples contained inconclusive descriptions such as *Normal Karyotype*, *Other*, *Complex Karyotype,* or relied on unclassified fusions or morphological descriptions with ambiguous equivalent WHO 2022 diagnoses such as in FAB=M0, M1, M6, and M7 (Supplementary Figure 2).

#### Diagnostic overall performance:

Overall model performance was also quantified using ROC curves, which showed minimum individual AUC values of 0.99 for the discovery cohort and 0.94 for the validation cohort, indicating excellent classification capability (Figure 2E,F). Other performance metrics for the training set included an accuracy of 0.963, macro F1 score of 0.948, weighted F1 score of 0.963, and Cohen’s Kappa of 0.96. In the validation set, the classifier achieved an accuracy of 0.901, a macro F1 score of 0.46, weighted F1 score of 0.94, and Cohen’s Kappa of 0.859 (Figure 2G).

#### Interactive features:

To facilitate data navigation and interpretation, we developed an interactive visualization tool for ALMA and provided it in the form of an open-source web-app: https://f-marchi.github.io/UF-LambaLab-ALMA-app/. This tool enables users to zoom, select, drag, and save specific areas of ALMA, as well as change tabs to compare the data under different clinical annotations. They can also select a particular cluster and observe its predicted risk profile. As a case study, we describe the *AML with t(v;11q23); KMT2A-r* cluster with samples colored according to WHO 2022 diagnosis, *ALMA Subtype*, and vital status (Supplementary Fig. 3). We also incorporated the newly reported expanded risk group for COG-AAML1831 trial^10^.

### Development of Epigenomic AML models of prognostic relevance

We used two approaches to develop models predictive of clinical outcome in pediatric AML (946 patients from discovery and 200 from validation cohort):

#### i) PaCMAP-LGBM-based prognostic model composed of 331556 CpGs:

**Figure 3:**
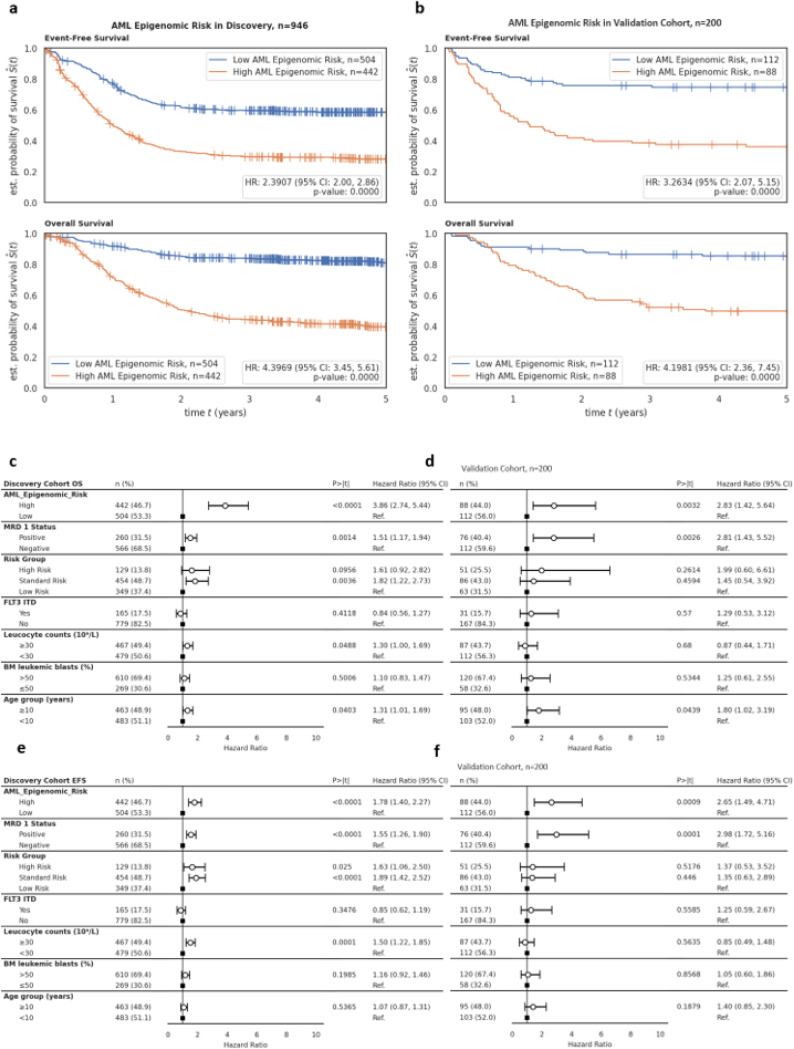
Patient outcomes and multivariate analyses of AML Epigenomic Risk groups. Patient outcomes by *AML Epigenomic Risk* groups in **A)** discovery cohort and **B)** validation cohort with EFS (top) and OS (bottom) of *AML Epigenomic Risk*^high^ (orange) and *AML Epigenomic Risk*^low^ (blue). Multivariate analysis adjusting for other confounding variables of OS in **C)** discovery cohort and **D)** validation cohort and EFS in **E)** discovery cohort and **F)** validation cohort. MRD 1, minimal residual disease at end of first induction; FLT3 ITD, FMS-like tyrosine kinase-3 internal tandem duplication; CI, confidence interval; Ref., reference.

The prognostic model herein named *AML Epigenomic Risk* is again a LightGBM supervised binary classifier developed using the 331556 CpGs compressed into five PaCMAP coordinates from ALMA to predict probability of death within 5 years for AML patients. In the discovery cohort (n=946), *AML Epigenomic Risk*^high^ demonstrated a markedly poorer OS (HR=4.40; 95% CI=3.45, 5.61; P<0.0001) and EFS (HR=2.39; 95% CI=2.00, 2.86; P<0.0001) in comparison to *AML Epigenomic Risk*^low^ (Figure 3A). In the validation cohort (n=200), patients within *AML Epigenomic Risk*^high^ group had poor OS (HR=4.20 95% CI=2.36, 7.45; P<0.0001) and EFS (HR=3.26; 95% CI=2.07, 5.15; P<0.0001) as compared to the *AML Epigenomic Risk*^low^ group (Figure 3B). Distribution of patient characteristics by *AML Epigenomic Risk* groups in discovery and validation cohorts is summarized in Supplementary Table S3. *AML Epigenomic Risk* was also significantly associated with induction I MRD in discovery and validation cohorts. *AML Epigenomic Risk* remained as an independent predictor of OS (HR=3.86; 95% CI=2.74, 5.44; P<0.0001) (Figure 3C) and EFS (HR=1.78; 95% CI=1.40, 2.27; P=0.0009) (Figure 3D) in multivariable analysis after adjusting for MRD1 status, risk group, FLT3 status, leucocyte counts at diagnosis, BM blast % at diagnosis, and age groups in discovery cohort. Similarly, in the validation cohort, *AML Epigenomic Risk* remained an independent predictor of OS (HR=2.83; 95% CI=1.42, 5.64; P=0.0032) (Figure 3E) and EFS (HR=2.65; 95% CI=1.49, 4.71; P=0.0009) (Figure 3F)

##### Prognostic model performance by risk group:

Given standard risk group patients prior to MRD results show significant variability in outcome despite lack of low or high-risk group features, we further evaluated *AML Epigenomic Risk* performance in standard risk group only. Within 229 standard risk group patients at study entry from the discovery cohort, patients with *AML Epigenomic Risk*^high^ had significantly poorer OS and EFS as compared to those with *AML Epigenomic Risk*^low^ (OS HR=4.37; 95% CI=2.42, 7.89; P<0.0001; EFS HR=2.00; 95% CI=1.36, 2.93; P=0.0004). Similar results were observed in the 86 standard risk patients from validation cohort (OS HR=4.95, 95% CI=1.88, 13.05; P=0.0012; EFS HR=2.08; 95% CI=1.07, 4.04; P=0.0304). Kaplan-Meier survival plots for EFS sand OS by low, standard, and high-risk group categories are shown in Supplementary Figure 4.

#### ii) An EWAS-CoxLasso-driven prognostic model composed of 38CpGs:

**Figure 4:**
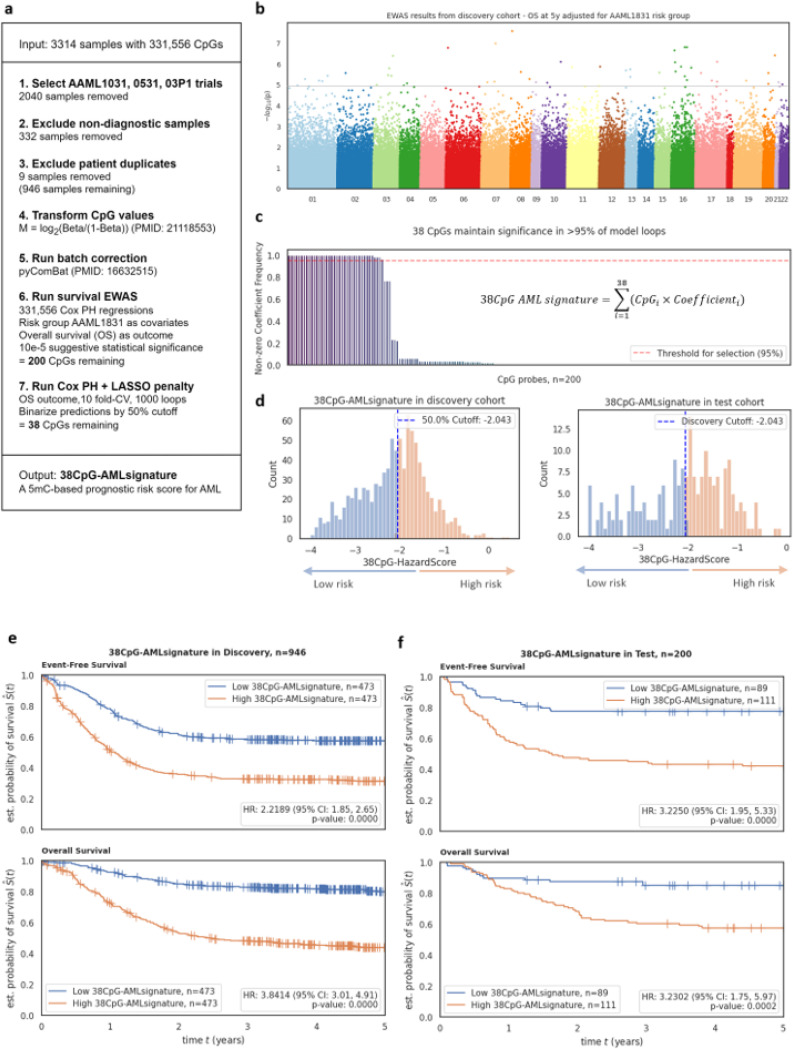
Development and testing of 38CpG AML signature. **A)** Stepwise workflow for generating the *38CpG-AMLsignature* model, including data preprocessing, batch correction, survival analysis, and penalized Cox PH modeling, resulting in a concise 5mC-based prognostic risk score for AML. **B)** Manhattan plot showing EWAS results with the significance of CpG probes across the genome, highlighting 200 CpGs that remained significant after p<10e-5 threshold. **C)** Stability analysis of 200 selected CpGs, showing the frequency of non-zero coefficients across 1000 model loops, which led to 38 CpGs being selected. **D)** Distribution of 38CpG-HazardScores in the discovery (left) and test (right) cohorts, dividing patients into high and low risk based on a 50% cutoff. **E)** Kaplan-Meier survival curves for EFS (top) and OS (bottom) in the discovery cohort (left, n=946) and **F)** validation cohort (right, n=200), comparing *38CpG-AMLsignature*^high^ (orange) and *38CpG-AMLsignature*^low^ (blue) groups.

As an alternative approach to generate a concise, time-to-event-based signature of prognostic relevance, a Cox-PH-regression-based EWAS was performed using 331556 CpGs in the discovery cohort (n=946) (Figure 4A). EWAS adjusted for risk group assignment identified 200 CpGs associated with 5-year time-to-death at a suggestive p-value threshold of 10e-5 (Figure 4B). We chose OS as our primary outcome measure since EFS definitions are not consistent across the various trials described here.

**Figure 5:**
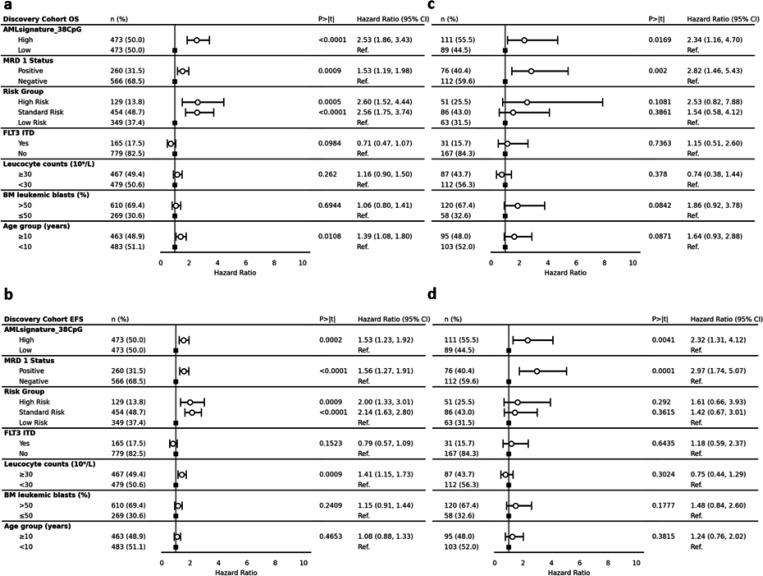
Forest plots of 38CpG AML signature against known confounding factors. **A)** Discovery cohort by OS. **B)** Discovery cohort by EFS. **C)** Validation cohort by OS. **D)** Validation cohort by EFS.

The 200 CpGs identified in the EWAS were evaluated using a penalized Cox-PH model with 1,000 iterations of 10-fold CV. Thirty-eight out of 200 CpGs consistently had non-zero coefficients in at least 95% of penalized fitting iterations (Figure 4C). The *38CpG-AMLsignature* was thus defined by the linear combination of parameters calculated from the CpG M-values applied to discovery cohort and validation cohorts. Details on the coefficients, CpGs from EWAS, their corresponding genomic loci, and overlapping genes are described in Supplementary Table S4. To devise a binary score, median cutoff of −2.043 was used, which allowed patients to be further categorized into high and low risk groups (Figure 4D). In the discovery cohort, patients with *38CpG-AMLsignature*^high^ had significantly poorer OS (HR=3.84; 95% CI=3.01, 4.91; P<0.0001) and EFS (HR=2.22; 95% CI=1.85, 2.65; P<0.0001) in comparison to *38CpG-AMLsignature*^low^ group (Figure 4E). In the validation cohort, consistent results were observed, where patients within *38CpG-AMLsignature*^high^ group had significantly poorer OS (HR=3.23; 95% CI=1.75, 5.97; P=0.0002) and EFS (HR=3.22; 95% CI=1.95, 5.33; P<0.0001) as compared to the *38CpG-AMLsignature*^low^ group (Figure 4F). Patient characteristics by *38CpG-AMLsignature* groups in discovery and validation cohorts are summarized in Supplementary Table S5 and show association with MRD after induction 1. Multivariable analysis in the discovery cohort after adjusting for MRD1 status, risk group, FLT3 status, leucocyte counts at diagnosis, BM blast % at diagnosis, and age groups posed the signature as an independent predictor of OS (HR=2.53; 95% CI=1.86, 3.43; P<0.0001) (Figure 5A) and EFS (HR=1.53; 95% CI=1.23, 1.92; P=0.0002) (Figure 5B, Supplementary Table S5). Finally, multivariable analysis in the validation cohort after adjusting for the same confounding factors also validated the signature as an independent predictor of OS (HR=2.34; 95% CI=1.16, 4.70; P=0.017) (Figure 5C) and EFS (HR=2.32; 95% CI=1.31, 4.12; P=0.004) (Figure 5D).

##### Signature performance by risk group:

As described above for *AML Epigenomic Risk*, here we analyzed the signature in standard risk group only. Within 229 standard risk group patients from discovery cohort, patients with *38CpG-AMLsignature*^high^ had significantly poorer OS and EFS as compared to those with *38CpG-AMLsignature*^low^ (OS HR=4.49; 95% CI=2.00, 6.08; P<0.0001; EFS HR=1.46; 95% CI=1.01, 2.11; P=0.0426). Similar results were also observed in the 86 standard risk patients from validation cohort (OS HR=3.08, 95% CI=1.07, 8.89; P=0.0374; EFS HR=2.57; 95% CI=1.14, 5.82; P=0.0233). Kaplan-Meiers survival plots for EFS and OS by low, standard, and high-risk group categories are shown in Supplementary Figure 5.

### AML Epigenomic Risk vs. 38CpG AML signature vs. standard of care

#### Comparative performance analysis of prognostic models:

Here we evaluated the predictive 5-year OS performance of *AML Epigenomic Risk*, *38CpG-AMLsignature,* and clinical trial risk groups. In ROC analysis of the discovery cohort, the AUCs were 0.71, 0.69 and 0.67 for *AML Epigenomic Risk*, *38CpG-AMLsignature* and the risk-group, respectively. In the validation cohort, the AUCs were 0.71, 0.66 and 0.70 for *AML Epigenomic Risk*, the *38CpG-AMLsignature* and risk-group, respectively (Supplementary Fig 6A). Pearson’s correlation between *AML Epigenomic Risk* and *38CpG-AMLsignature* was 0.7405 (p<0.0001) in the discovery cohort and 0.6859 (p<0.0001) in the validation cohort (Supplementary Fig 6B). These results were also summarized in confusion matrices, showing predicted vs. observed labels in discovery and validation cohorts for *AML Epigenomic Risk* model and *38CpG-AMLsignature* (Supplementary Fig 6C). Overall, *AML Epigenomic Risk* is superior to *38CpG-AMLsignature*, but it requires whole epigenome data (331556 CpGs) as compared to *38CpG-AMLsignature*, which offers a simple and concise solution (38 CpGs). Both models significantly improve upon current risk group classification for AML patients.

Given transcriptomic based leukemic stemness and drug resistance scores has been recently reported to have prognostic relevance ^11,12^, thus we compared the *AML Epigenome Risk* and *38CpG-AMLsignature* in context of these previously reported scores. As shown in Supplementary Figure 7, both epigenomic scores add additional predictive value beyond previously reported pLSC6 score^11,12^ (EFS and OS by *AML Epigenome Risk* within low Supplementary Figure 7A or high pLSC6 score groups Supplementary Figure 7B and *38CpG-AMLsignature* Supplementary Figure 7C and 7D. Corresponding ROC curves in discovery and validation cohorts for current signatures in context of previous transcriptomic signatures are also shown in Supplementary Figure 7E, and further bootstrap based Cox model shows similar results Supplementary Figure 7F. Similar results were observed in validation cohort Supplementary Figure 7G and 7H.

### A long-read specimen-to-result protocol for epigenomic diagnosis and prognosis of AML

A cohort of acute leukemia patients from adult and pediatric hematology/oncology units from UF Health Shands Hospital was evaluated in-house using a rapid long-read nanopore sequencing protocol to assess the rigor of our proposed models prospectively from specimen collection to result generation. A total of 12 specimens from 8 patients were processed, comprising of BM and/or PB from patients ranging in age from infants (1.25 years) to elderly adults (69 years).

**Figure 6:**
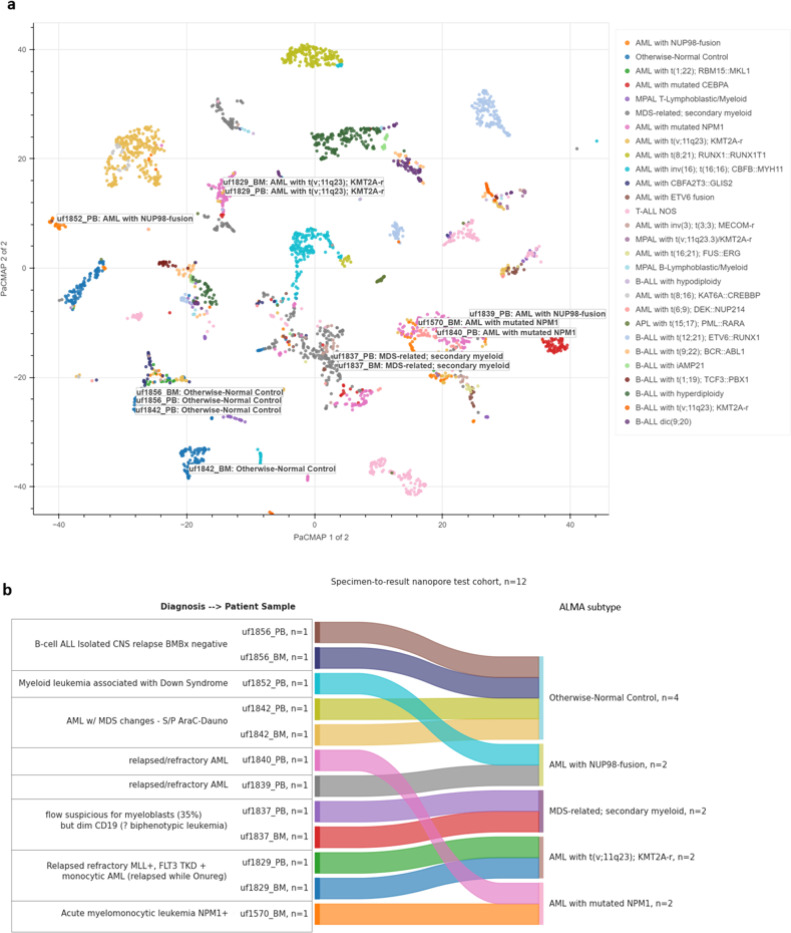
Specimen-to-result prospective testing using rapid nanopore long-read sequencing. **A)** Nanopore test cohort samples (n=12) mapped onto Acute Leukemia Methylome Atlas (ALMA) based on the combined methylation values of 331,556 CpGs. Each point represents a patient sample colored by ALMA Subtype. Samples with similar epigenomes cluster together, forming the island patterns seen through the scatterplot. Patient UF1829 was misplaced in two dimensions, but correctly placed in five dimensions. **B)** Sankey plot comparing clinical diagnosis information provided at sample collection (left) with ALMA Subtype classifier predictions (right) for the nanopore test cohort. The flow lines illustrate the correspondence between initial diagnostic classifications and the predicted ALMA subtypes. NUP98-fusion, Nucleoporin 98 fusion; PB, Peripheral Blood; BM, Bone Marrow; MDS, Myelodysplastic Syndrome; S/P: Status Post; Flow, Flow cytometry; TKD, Tyrosine Kinase Domain; CNS, Central Nervous System; BMBx, Bone Marrow Biopsy; NPM1, Nucleophosmin 1.

Time from specimen acquisition to sequencing start was 2h. Basecalling, modcalling, and alignment was primarily completed in 22h using a 150–300ng high molecular weight (HMW) DNA library loaded onto one PromethION flow cell per sample. 5mCG DNA modifications were collected for all sites in hg38 (28×10e6 CpGs) with >5x coverage but were subsequently filtered to the genomic loci of ALMA (331556 CpGs). Any missing values were imputed using the corresponding CpG mean of discovery cohort. PaCMAP model parameters were then applied to the data, which mapped each sample onto ALMA (Figure 6A). Finally, the *ALMA Subtype* model was applied and if subtype prediction matched with AML or MDS with >50% confidence, then *AML Epigenomic Risk and 38CpG AML signature* models were also applied and recorded.

### Predictions of ALMA Subtype match and refine preliminary pathology.

Model predictions closely matched preliminary pathology findings (Figure 6B). UF1570 (BM) was diagnosed with acute myelomonocytic leukemia NPM1+, and the predicted subtype was *AML with mutated NPM1* (0.885). UF1829 (BM and PB) presented with relapsed refractory monocytic AML with MLL fusion, and both samples were predicted as *AML with t(v;11q23); KMT2A-r* (BM: 0.583, PB: 0.986). UF1837 (BM and PB) had an ambiguous flow cytometry panel with 35% myeloblasts and dim CD19 expression, which prompted concern for biphenotypic leukemia; predictions classified both samples as *MDS-related; secondary myeloid* with high confidence (BM: 0.827, PB: 0.891). UF1839 (PB) presented with relapsed/refractory AML had a predicted subtype was *AML with NUP98 fusion* (0.553). UF1840 (PB), also presented with relapsed/refractory AML, was predicted as *AML with mutated NPM1* (0.551). UF1852 (PB) was diagnosed with myeloid leukemia associated with Down Syndrome and predicted as *AML with NUP98 fusion* (0.957). UF1856 (BM and PB) presented with isolated CNS relapse after treatment for B-cell ALL, and both samples were predicted as *otherwise-normal controls* (BM: 0.992, PB: 0.978), confirming negative bone marrow biopsy findings by pathology. Importantly, all patients who provided PB-BM pairs (UF1829, UF1837, UF1842, UF1856) had equivalent clinical subtype predictions despite tissue differences.

### AML Epigenomic Risk predictions.

Probability of death at 5 years according to AML Epigenomic Risk model for each patient was as follows: UF1570 (BM) had a probability of 0.424. UF1829 had a probability of 0.759 for BM and 0.754 for PB. UF1837 had a probability of 0.623 for BM and 0.711 for PB. UF1839 (PB) had a probability of 0.539. UF1840 (PB) had a probability of 0.424. UF1852 (PB) had a probability of 0.577. UF1856 was considered normal, so model results were not applicable. These risk predictions demonstrate the consistent assessment of prognosis across BM and PB samples, indicating that PB can effectively reflect the risk profile associated with AML and related subtypes.

### Final ALMA dataset enables analysis of generative population models (GPM).

We employed GPM to demonstrate the statistical rigor of ALMA, as well as its potential for new modalities of hypothesis-testing/generating research. First, certain joint distributions of our learned generative model were plotted against their empirical counterparts from the real dataset (Supplementary Fig. 8A). The inferred distributions qualitatively matched their empirical counterparts. Even after conditioning the model, joint distributions of variables agreed with their empirical counterparts (Supplementary Fig. 8B). For illustration, we have conditioned the learned model on race. As our inferred generative models are represented as probabilistic programs, we can transform and inspect them to search for complex non-linear relationships among variables. For instance, we estimated mutual information between the dimensionality reduced methylome variables PaCMAP1-PaCMAP5 (Supplementary Fig. 8C). Finally, our probabilistic program representation also allowed for further analyses of mutual information that could potentially reveal causal patterns.

## DISCUSSION

Genomic heterogeneity of myelogenous leukemia is proving to be far greater than the list of lesions currently present in diagnostic and prognostic guidelines. Currently, a third of patients at diagnosis are allocated to an ambiguous standard risk group, and a significant number in high- and low-risk are misplaced. These patients, if characterized adequately, could benefit from earlier transplantation, closer follow-up, and other treatment modalities. To address that, many studies identified DNA methylation and machine learning as promising avenues to improve diagnosis and prognosis in AML^4,13–16^. However, due to a lack of successful clinical implementation, these promising findings have yet to be practice changing. Here, we unveil a specimen-to-result clinical workflow for diagnosis and prognosis of AML using epigenomics, unsupervised learning, and single-molecule nanopore sequencing.

We describe the development of ALMA, the largest publicly available map of acute leukemia heterogeneity created by compressing methylation levels of 331556 CpGs from 3314 high-quality patient samples. ALMA enabled the creation of *ALMA Subtype*, a supervised classifier of 27 clinical WHO 2022 diagnostic subtypes plus otherwise-normal control. Additionally, we develop two models of prognostic relevance that are predictive of clinical outcome: a global *AML Epigenomic Risk* model using ALMA that predicts risk of death within 5 years, and an EWAS-CoxPH-based model named *38CpG-AMLsignature* that predicts mortality within 5 years using DNA methylation levels of 38 CpGs. These tools are made accessible to clinicians by way of a novel specimen-to-result protocol that uses 300uL of PB or BM to deliver simultaneous whole genome and epigenome sequencing, allowing for adjunct integration of our custom models with standard-of-care genomic assays. As proof of concept, we applied the protocol prospectively to 12 patient samples from Hematology/Oncology adult and pediatric inpatient units. Epigenomic model predictions were all concordant with preliminary pathology, equivalent between PB-BM pairs, and completed within 24h.

Our efforts reveal that most structural variations or recurrent mutations elicit a distinct epigenomic profile that matches closely with diagnostic subtyping and risk stratification. Regardless of the extent of the genomic insult — from single nucleotide variants to major structural variants — the resulting phenotype may be unambiguously identifiable at the epigenomic layer. Such findings have been described in other domains: A seminal study conducted in central nervous system tumors combined DNA methylation array data with machine learning tools to conclusively classify patients who might otherwise be misdiagnosed by standard of care methods^17,18^. These findings were clinically validated and shown to impact neurosurgical strategy^19^. Though these techniques have been successfully applied to other malignancies like sarcomas^6^, it is the first time that they are comprehensively described in the hematopoietic system.

From a computational biology perspective, we aimed to set a new standard of transparency and rigor by creating a step-by-step electronic notebook with source code, explanation, and resulting figures for all analyses undertaken in this study. This includes a custom interactive interface for ALMA (https://f-marchi.github.io/UF-LambaLab-ALMA-app/), which allows for broad scrutiny of our data and further study of its underlying biology. Of note, adding ALL, APL, MPAL, MDS, and control samples of patients of all ages to ALMA led to increased classification accuracy of AML subtypes and especially increased prediction accuracy for very rare subtypes, like MECOM-r, without compromising others. Importantly, it led to *ALMA Subtype* providing diagnosis of samples containing rare recurring mutations or complex karyotypes, which are notoriously challenging for current standard of care approaches like FISH, PCR, and short-read sequencing panels.

Given that prognosis is a function of diagnostic subtype and standard of care, the clusters present in ALMA also translate to prognostic classification, evidenced by the ability of *AML Epigenomic Risk* to serve as a substantial predictor of overall survival with robust testing results in fully independent trials. This is not only due to the algorithms and datasets used, but also due to the stable nature of DNA methylation, which is covalently bound to double-stranded DNA and hence a remarkably inert marker of cellular differentiation and tissue of origin. Two prognostic models were created because testing for limited numbers of CpGs may allow for broader utility where whole genome sequencing is unavailable. Our EWAS-CoxPH based stepwise approach resulted in a *38CpG-AMLsignature* with similar predictive capacity as *AML Epigenome Risk*,allowing centers and investigators to choose between the two options according to their resources. 11,1211,12

We chose long-read sequencing for clinical implementation since it is the only technology presently capable of detecting most structural variants, repeats, and DNA modifications out of a single run. Furthermore, 5mC calling is done natively at a single-molecule level without bisulfite conversion and amplification bias. However, costs per genome are still prohibitive, which begs for the development of more competitive and innovative platforms and strategies. Ultimately, we compared our epigenomic models to preliminary pathology findings for twelve patient samples collected and processed from specimen to result at UF Health Hospital. Our findings prospectively showed diagnostic concordance for all samples. Furthermore, the predictions for paired BM and PB samples from the same patients were all equivalent, despite tissue variability. However, further studies in larger cohorts are needed to validate these observations, especially in notoriously elusive subtypes like AML with CBFA2T3::GLIS2 fusions.

In conclusion, this study introduces a clinical workflow that utilizes nanopore epigenomic sequencing for sensitive and specific diagnosis and prognosis of AML patients. We aimed to map the heterogeneous landscape of acute leukemias by way of the unsupervised ALMA foundational model. And finally, we introduced three robust algorithms: *ALMA Subtype*, *AML Epigenomic Risk*, and *38CpG-AMLsignature*, all of which showed state-of-the-art diagnostic or prognostic capacity, respectively. These efforts highlight the potential for increased affordability, speed, and accuracy in oncology care and trial design, ultimately benefitting of patients worldwide.

## ONLINE METHODS

### Patient characteristics

In this study, we assembled and harmonized raw data from publicly available methylation datasets of acute leukemias from 11 clinical trials/studies, making it one of the largest cohorts to be evaluated. Supplementary Table S1 provides details on these 11 cohorts: NOPHO ALL92–2000 (n=933)^20^, AAML0531 (n=628)^21,22^, AAML1031 (n=587)^23,24^, BeatAML (n=316)^16^, TCGA AML (n=194)^25^, French GRAALL 2003–2005 (n=141)^26^, TARGET ALL (n=131)^27^, CETLAM SMD-09 (n=166)^28^, AAML03P1 (n=72)^29^, Japanese AML05 (n=64)^30^, and CCG2961 (n=41)^31^, resulting in a total of 3,314 patients after preprocessing and quality control assessment. Samples were obtained either from bone marrow or peripheral blood at diagnosis, relapse, or remission. DNA methylation data was procured using the Illumina methylation array 450k or EPIC array, which share 452,453 probes with the same chemistry and design.

For the development of AL Epigenomic Phenotype, our fine-tuned supervised diagnostic model, a subset of 2471 samples were selected for having WHO 2022 diagnostic annotation data (Table S1). Clinical annotations such as karyotype, cytogenetics, gene fusion, or otherwise-specified diagnostic information were used to create European LeukemiaNet (ELN) and World health Organization (WHO) 2022 subtypes.

For the development of both the models of prognostic relevance, we restricted our analysis to specimens obtained at diagnosis (predominantly bone marrow aspirates) from the Children’s Oncology Group (COG) trials AAML1031 (NCT01371981), AAML0531 (NCT00372593) and AAML03P1 (NCT00070174) through GSE190931, GSE124413 and GDC_TARGET-AML datasets, respectively. The clinical endpoints were defined as: i) Minimal residual disease after induction 1 (MRD1): positive MRD1 if ≥ 1 leukemic cell per 1,000 mononuclear bone marrow cells (≥ 0.1%) determined by flow cytometry; ii) event-free survival (EFS) defined as the time from study enrollment to induction failure, relapse, secondary malignancy, death, with event-free patients censored on last follow-up; iii) overall survival (OS) defined as the time from study enrollment to death, with living patients censored on the date of last follow-up.

To independently test the findings derived from the discovery cohort, we processed in parallel DNA methylation data from 200 AML patients treated on the multi-center clinical trials AML02 (NCT00136084) and AML08 (NCT00703820) ^32,33^.

### Data quality control and preprocessing

SeSAMe^34^, which is the selected methylation processing software of National Cancer Institute’s Genomic Data Commons and COG^28^ was utilized for preprocessing of the raw methylation array ^35^. Preliminary quality control exclusion criteria included: i) 12003 sex-linked and non-CpGs; ii) 47382 CpGs deemed unreliable based on literature benchmarking^36^; iii) 460 samples due to Illumina quality control p-value failure; iv) 61512 CpGs with over 5% missing values; v) 60 non-hematopoietic samples; vi) 11 samples considered outliers according to PCA analysis. Finally, interpolation by batch mean filled the remaining missing values^37^. To adjust for potential confounding variability, batch correction was performed using *ComBat*^38,39^. The final dataset of 331556 CpGs and 3314 samples was considered for downstream statistical analyses (Supplementary Fig 9).

### Development of custom models of diagnostic and prognostic relevance:

#### Acute Leukemia Methylome Atlas (ALMA):

To capture the heterogeneous landscape of acute leukemia by epigenomics, we used a dimensionality reduction algorithm called Pairwise Controlled Manifold Approximation (PaCMAP)^40^, which allowed compression of processed CpG β-values into two dimensions for visualization and five dimensions for supervised classification analysis.

#### ALMA Subtype:

To devise the diagnostic classifier, we used five PaCMAP dimensions (coordinates), which represent the combined compressed values for 331556 genome-wide CpGs. These were input to Light Gradient Boosting Machine (LGBM), a decision-tree based supervised learning algorithm. Hyperparameter tuning using 5-fold cross validation (CV) involved Lasso and Ridge penalties with balanced class weights to account for rarer subtypes. For diagnosis, we assessed accuracy per class of 27 WHO subtype categories plus otherwise-normal control (a one-vs-rest multi-class classifier of 28 clinical subtypes). Not all samples, however, had WHO 2022 annotation available in the dataset to serve as ground truth, so only those with annotations were used (n=2471 discovery cohort; n=104 validation cohort). The *otherwise-normal control* category is composed of samples described as “normal” or believed to be without disease. These are normal bone marrow or peripheral blood samples from either otherwise-healthy subjects or patients in remission and without evidence of disease. No cell lines or sorted primary cells were used as controls.

#### AML Epigenomic Risk:

To devise the prognostic classifier, 946 AML-only PB or BM samples at diagnosis were used. These were selected from the Children’s Oncology Group (COG) trials AAML1031 (NCT01371981), AAML0531 (NCT00372593) and AAML03P1 (NCT00070174) through GSE190931, GSE124413 and GDC_TARGET-AML datasets, respectively. The same strategy listed as above was used, where five PaCMAP dimensions were input to a LGBM classifier with hyperparameter tuning in 5-fold CV. The target output, however, was OS at 5 years (dead/alive status). Testing was performed in an independent dataset of 200 AML patients from multi-center AML02 and AML08 trials.

#### 38CpG-AMLsignature:

As an alternative approach and to devise a concise epigenomic signature of prognostic relevance, we used the same samples as described above. Using 331556 CpGs, we conducted an epigenome-wide association study (EWAS) using Cox Proportional Hazards regression to identify CpGs with methylation levels most predictive of OS at 5 years while adjusting for risk group categories. Next, we selected CpGs at suggested p-value threshold of 10e-5 as input to LASSO-based multivariate Cox-PH model, with 1000 iterations of 10-fold cross-validation. CpGs selected in 95% of the models were utilized to create an epigenomic signature. To create a binary risk score, patients were further categorized into either *38CpG-AMLsignature*^low^ or *38CpG-AMLsignature*^high^ groups by median cutoff.

### Software tools and source code

Source code for all analyses and figures, as well as diagrams and resulting plots are publicly available at the electronic notebook we created for this study: https://f-marchi.github.io/ALMA/. Software and hardware information from each analysis are noted at the end of each chapter of the under section “Watermark”. Of note, methylation array preprocessing software used was *methylprep* v.1.7.1 in python 3.7, followed by *methylcheck* v0.8.5 in python 3.8. Statistical learning analyses were implemented with *scikit-learn* v1.2.2*, scikit-survival* v0.20.0, *statsmodels* v0.13.5, and *lifelines* v0.27.7^41–43^. Patient characteristics table used *tableone* v0.7.12^44^. Plotting packages were *matplotlib* v3.7.1, *bokeh* v3.3.4, and *seaborn* v0.12.2^45–47^. Data structure and manipulation packages were *numpy* v1.24.4 and *pandas* v2.0.3^48,49^. Sankey plots were adapted from *pySankey* v0.0.1. Finally, *pacmap* v0.7.0 and *lightgbm* v3.3.5 were used^50^.

### Machine learning rigor, clarity, and reproducibility

To abide by the criteria recently proposed in the literature of machine learning applications in life sciences and establish the rigor of our bioinformatic pipelines^51^, we made the raw training data, processed training data, model weights, and source code publicly available and open source. All methods and results from this study were written in step-by-step format using Jupyter Book through GitHub-pages. Additionally, testing samples come from independently conducted clinical trials and independently processed pipelines. Specimen-to-result testing was done in-house using nanopore sequencing to unequivocally validate findings in distinct platforms.

### Data Availability

Discovery (training) data analyzed in this study were obtained from Gene Expression Omnibus (GEO) at GSE190931, GSE124413, GSE133986, GSE159907, GSE152710, GSE49031, GSE147667, as well as from Genomic Data Commons (GDC) at GDC_TARGET-AML, GDC_TCGA-AML, GDC-TARGET-ALL. Patient-level data from nanopore, discovery, and validation cohorts can be found in Supplementary Table S2 along with each patient’s available clinical data.

### Specimen-to-result testing protocol

#### High-molecular-weight DNA extraction:

Whole blood or bone marrow (300 μL) samples in EDTA were processed using a modified QIAGENE Puregene Blood Core Kit protocol adapted from Goenka et al ^52^. Briefly, RBC lysis in clot-free samples followed by centrifugation at 16,000 x g for 1 minute was used to pellet white blood cells. Cell lysis was achieved by adding 600 μL of Cell Lysis Solution, pipetting, and vortexing at 3000 rpm for 20 seconds. RNase A Solution (1.5 μL) was added, and the mixture was inverted 25 times before incubation at 56°C for 5 minutes. Protein precipitation was facilitated by adding 200 μL of Protein Precipitation Solution, followed by incubation on ice for 5 minutes and centrifugation at 16,000 x g at 4°C for 4 minutes. The supernatant was transferred to a tube containing 600 μL of isopropanol, gently flipped 50 times, and centrifuged at 16,000 x g for 1 minute to pellet the DNA. The DNA pellet was washed with 600 μL of 70% ethanol, centrifuged, and dried at 37°C for 2 minutes before being dissolved in 100 μL of DNA Hydration Solution and incubated at 65°C for 10 minutes.

#### DNA shearing and quantification:

DNA samples were sheared using a 26G 0.5in needle attached to a 1 mL Luer slip tip syringe. The needle was placed at the bottom of a 1.5 μL DNA LoBind sample tube, and the liquid was drawn up to the 0.3 mL line and expelled back into the tube. This shearing process was repeated five times to achieve the N50 of 20kb. For DNA quantification, the Qubit dsDNA BR Assay was used according to the manufacturer’s protocol in triplicate measurements.

#### Library preparation and sequencing:

For sequencing, the Rapid Sequencing Kit V14 - gDNA (SQK-RAD114) was used as per manufacturers protocol with the following modifications: i) input mass was increased to 300ng; ii) volume of fragmentation mix (FRA) and rapid adapter (RAP) doubled to 2uL. Prepared library was loaded into a PromethION 2 (P2) Solo sequencer by slowly rotating pipet plunge.

#### Basecalling, modcalling, and alignment:

Raw signal data (POD5) collection was done using *MinKNOW* software and transferred. Basecalling was executed using Oxford Nanopore Technology (ONT) basecaller *Dorado* v0.5– v0.8 using high accuracy (hac) or super high accuracy (sup) model versions 4.3.0 and 5.0.0. The GPU used was one local NVIDIA GeForce RTX 4090. The reference genome used was *GCA_000001405.15_GRCh38_no_alt_analysis_set.fna* (hg38). Alignment was conducted using *Minimap2* using the *lr:hq* preset^53^. 5-methylcytosine-guanine (5mCG) modifications were called by ONT modcaller *remora*. Both mod calls and alignment were done through *Dorado* command line.

#### Long-read data processing:

Post-sequencing data processing included sorting BAM files using *samtools* v1.13^54^. The total number of reads, unmapped reads, total bases, and N50 values were reported alongside mean coverage on hg38 and median accuracy scores. Genomic variant analysis was done using *epi2me* v5.1.10–5.2.1 and *wf-human-variation* v2.0.0–2.4.1, which covered the following modalities and tools: single nucleotide variants (SNVs), structural variations (SVs), copy number variations (CNVs), short tandem repeats (STRs), and 5-methylcytosine (5mC) and 5-hydroxymethylcytosine (5hmC) modifications^55^. SNVs were identified with *Clair3* v1.0 and classified into pathogenic SNVs and drug response SNVs by *ClinVar*^56^. SVs, including inversions, duplications, and translocations, were detected using *Sniffles2* v2.0.7-epi2me^57^. CNVs were called with *QDNAseq* v1.34.0 and/or *Spectre* v0.2.2, specifying abnormalities such as gains and losses of chromosomes or chromosomal segments^58^. STRs were identified using *straglr-genotype* v1.4^59^. Finally, 5mC and 5hmC modifications tags were processed into bed files with *modkit* v0.2.5-v0.3.3.

#### Model testing on long-read DNA methylation data:

To test models using nanopore platform, we created a *pacmap_reference* BED file containing genome coordinates for 331556 CpGs according to hg38. For each nanopore sample, a BED file provided by *modkit* containing genomic coordinates for 28M CpGs is loaded and merged with *pacmap_reference*. DNA-methylation data structure is the same between Illumina arrays and nanopore sequencing since it represents the fraction of methyl groups encountered at a given CG locus, so no data transformation was necessary. The pipeline then proceeds as described in the test cohort.

### ALMA generative population models.

We aimed to validate ALMA using the framework of GPMs, which automate error-prone aspects of data cleaning and analysis. Briefly, a GPM consumes a set of sparsely overlapping datasets and infers through Bayesian structure learning^60^ a probabilistic program representing a generative model of the combined data (Supplementary Fig 10). This enables interaction with the learned generative program via natural language prompts semantically parsed and translated into queries in GenSQL^61^ and optimized and lowered to probabilistic programs in SPPL^62^. Moreover, the learned generative programs can generate synthetic datasets that match the statistical properties of the combined datasets while not revealing any sensitive patient information or suffering from missing values (Supplementary Fig 8A and 8B). Further, the probabilistic program representation facilitates causal analyses or poststratification (Supplementary Fig 8C)^63^.

## Supplementary Material

Supplement 1
