# Supplementary material for: Long-read epigenomic diagnosis and prognosis of Acute Myeloid Leukemia
Source: Res Sq. 2024 Dec 12:rs.3.rs-5450972. Preprint. [Version 1] doi: 10.21203/rs.3.rs-5450972/v1 (PMC11661290; doi:10.21203/rs.3.rs-5450972/v1)
Supplement: Supplement 1 [file NIHPPRS5450972V1-supplement-1.pdf]

Supplementary Files

This is a list of supplementary files associated with this preprint. Click to download.

- ALMAFiguresv19FM11.1324FMSupplementaryFigures.pdf
- SupplementartyTable1.xlsx
- SupplementaryTable2.xlsx
- SupplementaryTable3.xlsx
- SupplementartyTable4.xlsx
- SupplementaryTable51.pdf
